# VEGF Correlates with Inflammation and Fibrosis in Tuberculous Pleural Effusion

**DOI:** 10.1155/2015/417124

**Published:** 2015-03-26

**Authors:** Mauo-Ying Bien, Ming-Ping Wu, Wei-Lin Chen, Chi-Li Chung

**Affiliations:** ^1^School of Respiratory Therapy, College of Medicine, Taipei Medical University, Taipei 110, Taiwan; ^2^Division of Pulmonary Medicine, Department of Internal Medicine, Taipei Medical University Hospital, Taipei 110, Taiwan; ^3^Department of Obstetrics and Gynecology, Chi-Mei Medical Center, Tainan 710, Taiwan; ^4^Department of Obstetrics and Gynecology, College of Medicine, Taipei Medical University, Taipei 110, Taiwan; ^5^Graduate Institute of Medical Sciences and Department of Pharmacology, College of Medicine, Taipei Medical University, Taipei 110, Taiwan

## Abstract

*Objective*. To investigate the relationship among angiogenic cytokines, inflammatory markers, and fibrinolytic activity in tuberculous pleural effusion (TBPE) and their clinical importance. *Methods*. Forty-two patients diagnosed with TBPE were studied. Based on chest ultrasonography, there were 26 loculated and 16 nonloculated TBPE patients. The effusion size radiological scores and effusion vascular endothelial growth factor (VEGF), interleukin- (IL-) 8, plasminogen activator inhibitor type-1 (PAI-1), and tissue type plasminogen activator (tPA) were measured. Treatment outcome and pleural fibrosis, defined as radiological residual pleural thickening (RPT), were assessed at 6-month follow-up. *Results*. The effusion size and effusion lactate dehydrogenase (LDH), VEGF, IL-8, PAI-1, and PAI-1/tPA ratio were significantly higher, while effusion glucose, pH value, and tPA were significantly lower, in loculated than in nonloculated TBPE. VEGF and IL-8 correlated positively with LDH and PAI-1/tPA ratio and negatively with tPA in both loculated and nonloculated TBPE. Patients with higher VEGF or greater effusion size were prone to develop RPT (*n* = 14; VEGF, odds ratio 1.28, *P* = 0.01; effusion size, odds ratio 1.01, *P* = 0.02), and VEGF was an independent predictor of RPT in TBPE (receiver operating characteristic curve AUC = 0.985, *P* < 0.001). *Conclusions*. Effusion VEGF correlates with pleural inflammation and fibrosis and may be targeted for adjunct therapy for TBPE.

## 1. Introduction

Tuberculosis (TB) remains a major global public health issue and continues to cause significant morbidity and mortality worldwide [1]. Tuberculous pleural effusion (TBPE) is the most common form of extrapulmonary TB and often complicated with pleural fibrosis [2]. This pleural fluid is enriched in proteins, inflammatory cells, and various angiogenic cytokines [3], including vascular endothelial growth factor (VEGF) and interleukin- (IL-) 8, which stimulate migration of leukocytes, induce vascular hyperpermeability and pleural fluid production, activate coagulation cascade, and repress fibrinolytic activity within the pleural cavity [4, 5].

Fluid loculation with fibrin septation, a hallmark of pleural inflammation, is commonly found to be the initial presentation of TBPE [6]. Loculated effusion, defined as effusion that does not move freely in the pleural space due to fibrinous adhesion between parietal and visceral pleura, makes reabsorption or drainage of such fluid collection very difficult and leads to persistent dyspnea [6]. Fibrin turnover in the pleural cavity is affected by fibrinolytic activity mediated by plasmin, which is regulated by the equilibrium between plasminogen activators (PAs) and plasminogen activator inhibitors (PAIs) [7]. An imbalance between PAI-1 and tissue type plasminogen activator (tPA) may elicit fibrin gel formation in the pleural space and lead to pleural fluid loculation, fibrin neomatrix remodeling, and fibrosis [6, 8].

VEGF may facilitate the genesis of fibrin gel in infectious pleural effusions [9]. Previous studies reported that VEGF might play a role in the modulation of tPA and PAI-1 [10] and that anti-VEGF antibody could reduce fluid volume of inflammatory pleural effusion and attenuate pleural inflammation and fibrosis [11–13]. These findings suggest that VEGF may be involved in the regulation of inflammation, fibrin turnover and fluid loculation in the pleural cavity, and subsequent residual pleural thickening (RPT) or fibrosis [9], which was observed in our previous study on parapneumonic effusions [14]. However, the clinical relevance of angiogenic cytokines and fibrinolytic activity in TBPE remains unclear. The aim of the present study was to evaluate the relationship among angiogenic cytokines (VEGF, IL-8), inflammatory markers (lactate dehydrogenase (LDH), glucose, pH value), and fibrinolytic parameters (tPA and PAI-1) in TBPE and their clinical implication.

## 2. Methods

### 2.1. Study Design

This single-center prospective study intended to assess the clinical importance of angiogenic cytokines and fibrinolytic activity in TBPE. Ethics approval (CRC-05-11-01) was obtained from the Institutional Review Board of Taipei Medical University (Taipei, Taiwan), and all patients gave written informed consent before entering the study.

### 2.2. Patient Selection

Consecutive patients with pleural effusion (PE) of unknown cause admitted to Taipei Medical University Hospital were eligible for this study and were included when a diagnosis of TBPE was established by the demonstration of granulomatous pleuritis on closed pleura biopsy specimens with or without the presence of acid-fast bacilli. Exclusion criteria were as follows: history of invasive procedures directed into the pleural cavity; recent severe trauma, hemorrhage, or stroke; bleeding disorder or anticoagulant therapy; use of streptokinase in the previous 2 years; and lack of dyspnea caused by effusions.

### 2.3. Imaging of PE

All patients were subjected to routine chest radiography (CXR, frontal and lateral views), lateral decubitus view with the lesioned side down, real-time chest ultrasonography (US), and/or thoracic computed tomography (CT) to determine the loculated or nonloculated PE as previously described [6]. Loculated effusion was diagnosed if the fluid collection (1) appeared as a fixed lenticular shape on a frontal CXR and was unchanged in appearance on a decubitus CXR or (2) had a lobulated or lentiform configuration with a convex smooth border on chest US or CT imaging.

### 2.4. CXR Scoring

The posteroanterior CXR films were read and scored by two radiologists who were blind to any clinical information to determine (a) the largest linear width of pleural opacity and (b) effusion size CXR score: the estimated overall percentage of pleural shadowing in the hemithorax [15].

### 2.5. Thoracentesis and Pleural Fluid Analysis

With the guidance of chest US, 50 mL of pleural fluid was aspirated immediately or within 24 hours after hospitalization. When PE was multiloculated, the fluid was aspirated from the largest loculus. Pleural fluid analyses, adenosine deaminase (ADA), and microbiological studies were performed routinely.

### 2.6. Measurement of Effusion VEGF, IL-8, PAI-1, and tPA

The commercially available enzyme-linked immunosorbent assay kits were used to measure effusion levels of VEGF, IL-8 (R & D System; Minneapolis, MN, USA), tPA, and PAI-1 (American Diagnostica; Greenwich, CT, USA) as previously described [6].

### 2.7. Management of TBPE

Standard anti-TB medications in addition to pigtail drainage were administered once TB pleurisy was diagnosed. Intrapleural injection therapy started on the following day and was done once daily for three continuous days. Patients with nonloculated effusion underwent intrapleural injection with 50 mL normal saline. Patients with loculated effusions received intrapleural injection with solutions containing 50 mL normal saline with 250,000 IU of dissolved streptokinase (Aventis, Marburg, Germany). After injection, the pigtail tube was clamped for 2 hours and then opened for free drainage. CXR was performed after the third day of treatment. Complete drainage was defined as no or minimal pleural effusion on CXR. The pigtail tube was removed when the net drainage was less than 50 mL during the previous 24 hours.

### 2.8. Outcome Measures

CXR and pulmonary function testing with spirometry were performed on discharge and 6 months later, respectively. RPT was measured and defined as a lateral pleural thickening of ≥10 mm shown on CXR and confirmed by chest US or CT at the end of 6-month follow-up [16].

### 2.9. Statistical Analysis

Data were expressed as mean ± SD, median (interquartile range or range), or frequency (%), where appropriate. Comparisons of continuous data were made using an unpaired *t*-test or Mann-Whitney *U* test between two groups where appropriate. The correlations between variables were determined by Spearman rank correlation coefficients. Categorical variables between two groups were examined using *χ* ^2^ method and/or Fisher's exact test, when appropriate. A two-tailed *P* value <0.05 was considered to be statistically significant.

Multivariate logistic regression analyses were performed to determine factors independently associated with development of RPT. Variables found to be significant in the univariate analysis were entered into a binary logistic regression analysis. Results of multivariable analyses are reported as odds ratios (OR) with 95% confidence intervals and *P* values. The optimal sensitivity, specificity, and cutoff value of pleural fluid variables to predict RPT were evaluated by the receiver operating characteristics (ROC) by analyzing the area under the curve (AUC).

## 3. Results

### 3.1. Patient Characteristics

Consecutive 50 patients with TBPE were eligible for this study. Eight patients were excluded because of recent stroke in three, recent gastrointestinal bleeding in two, and informed consent unavailable in three cases, respectively. Finally, 42 patients were enrolled, including 27 men and 15 women with an age range from 22 to 91 years (mean age, 62 years) (Table 1), and all completed 6 months of follow-up from March 2011 through June 2014.

### 3.2. Comparisons between Loculated and Nonloculated TBPE

There were 26 patients with loculated TBPE and 16 patients with nonloculated TBPE (Table 1). Clinical data, pleural fluid characteristics, angiogenic cytokines, and parameters related to fibrinolytic activities in pleural fluids are shown in Table 2. Compared to patients with nonloculated TBPE, loculated TBPE patients had significantly higher effusion CXR score on admission. No significant differences between the two groups were found in terms of age, gender, comorbidities, and duration of illness before treatment. Patients with loculated TBPE had significantly higher levels of effusion LDH, VEGF, IL-8, PAI-1, and PAI-1/tPA ratio, and lower values of pH, glucose, and tPA than did nonloculated TBPE patients. Moreover, the ADA level, protein concentrations, and leukocyte counts were comparable between two groups.

### 3.3. Correlations among Effusion Angiogenic Cytokines, Fibrinolytic Parameters, Pleural Fluid Characteristics, and Effusion CXR Score

As shown in Table 3, the effusion levels of IL-8 and VEGF were positively correlated with those of LDH and PAI-1/tPA ratio and negatively correlated with those of tPA in both loculated and nonloculated TBPE. In addition, VEGF correlated positively with PAI-1 and negatively with pH value and glucose in both loculated and nonloculated TBPE, suggesting that VEGF is implicated in pleural inflammation and fibrinogenesis.

The effusion CXR score had significant positive correlation with the effusion levels of VEGF in both loculated and nonloculated TBPE. However, there was no significant correlation between the effusion size and the effusion levels of IL-8.

### 3.4. Comparisons between TBPE Patients with and without RPT

All patients were successfully treated with anti-TB medications and intrapleural instillation of normal saline or streptokinase and were discharged uneventfully. All patients finished the 6-month anti-TB medications and improved clinically over time, showing no recurrence of the disease. RPT was observed in 14 patients (33%) at the end of 6-month follow-up (Table 4). All of them (100%) had loculation of pleural effusions initially. The effusion CXR score on admission and the effusion levels of leukocyte count, PAI-1, PAI-1/tPA ratio, IL-8, and VEGF were significantly higher, and the effusion pH value and tPA were significantly lower in the patients with RPT than in those without RPT. Moreover, patients with RPT had significant lower forced vital capacity (FVC) than those without RPT.

### 3.5. Multivariate Logistic Regression Analysis

Furthermore, multivariate logistic regression analysis was used to identify the independent factors associated with RPTin TBPE after 6-month anti-TB medications (Table 5). Variables of significance in univariate analysis were included for analysis which demonstrated that only higher effusion VEGF level and greater effusion CXR score were independent predictors for RPT in TBPE.

### 3.6. Optimal Sensitivity, Specificity, and Cutoff Value of Variables to Predict RPT

**Figure 1:**
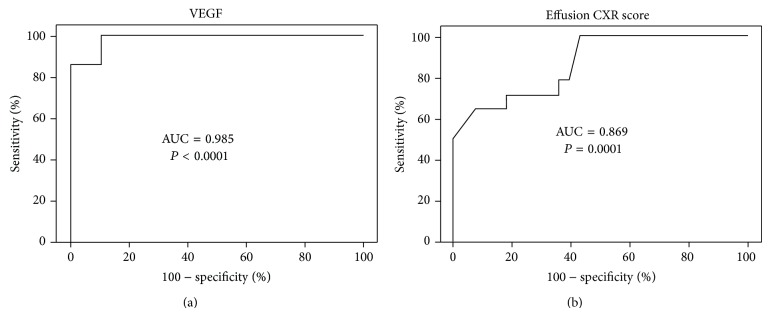
Receiver operating characteristic curves for (a) effusion vascular endothelial growth factor (VEGF) level and (b) effusion chest radiography (CXR) score to predict residual pleural thickening (RPT) in tuberculous pleural effusion (TBPE). AUC = area under the curve.

The ROC curve showed that the effusion VEGF at the cutoff level >842 pg/mL had the highest sensitivity and specificity for predicting RPT in TBPE patients (area under the ROC curve = 0.985, 95% CI = 0.957–1.012; sensitivity 100%, 95% CI = 76.8–100%; specificity 89.3%, 95% CI = 71.8–97.7%) (Figure 1(a)), followed by effusion CXR score >62% (area under the ROC curve = 0.869, 95% CI = 0.757–0.981; sensitivity 64.3%, 95% CI = 35.1–87.2%; specificity 92.9%, 95% CI = 76.5–99.1%) (Figure 1(b)).

## 4. Discussion

Our results demonstrated that effusion size, LDH, VEGF, IL-8, PAI-1, and PAI-1/tPA ratio were significantly higher, while effusion glucose, pH value, and tPA were significantly lower, in loculated than in nonloculated TBPE. VEGF and IL-8 correlated positively with LDH and PAI-1/tPA ratio and negatively with tPA in both loculated and nonloculated TBPE. Additionally, VEGF had positive correlation with effusion size in both loculated and nonloculated TBPE. Fourteen patients who developed RPT at the end of 6-month follow-up had larger effusion size and higher levels of VEGF and PAI-1/tPA ratio than those who did not. Moreover, VEGF and effusion size were independent predictors of RPT in TBPE. To our knowledge, this is the first study to demonstrate that VEGF correlated significantly with TB pleural inflammation and fibrinolytic activity and that elevated VEGF level was associated with development of pleural fibrosis in TBPE.

Previous studies showed that the level of VEGF was consistently higher in exudative than in transudative pleural effusions [17, 18] and TBPE contained significantly higher levels of VEGF than did pleural fluid of heart failure patient [19]. Another report demonstrated that compared to patients with inactive pulmonary TB and control subjects, serum VEGF levels were increased in patients with active pulmonary TB and were decreased after successful treatment, indicating that VEGF may serve as a marker of disease activity [20]. Likewise, the present study revealed that VEGF levels were significantly higher in loculated than in nonloculated TBPE and correlated substantially with pleural inflammatory markers such as LDH, pH, and glucose in both groups. As pleural inflammation and increased vascular permeability and leakage are essential for the development of exudative PE, VEGF may represent a key mediator in pleural fluid formation [21]. In parallel, our data disclosed a substantial correlation between effusion VEGF level and effusion CXR score in both loculated and nonloculated TBPE. All these results suggest that VEGF is implicated in pleural inflammation and may be crucial for the formation of TBPE.

VEGF induces extravascular leakage of plasma proteins and is important in the modulation of extracellular matrix proteolysis by regulating the expression of tPA and PAI-1 in endothelial cells [10]. Furthermore, VEGF has been reported to increase PAI-1 expression in keloid fibroblasts and to contribute to dermal fibrosis [22]. Another angiogenic factor IL-8 has been shown to increase vascular permeability and fluid exudation in endotoxin-induced pleurisy* in vivo* [23] and correlated positively with PAI-1 and negatively with tPA in exudative PE [24]. All these findings indicate that angiogenic cytokines may elicit exudative effusions and modulate fibrinolytic activity in pleural space by altering the balance of PAI-1 and tPA. In line with the previous reports [10, 22–24], our data demonstrated that in both loculated and nonloculated TBPE, the levels of VEGF and IL-8 correlated positively with the values of PAI-1/tPA ratio and negatively with tPA level, though only VEGF levels correlated positively with PAI-1 values. In addition, the levels of VEGF, IL-8, PAI-1, and PAI-1/tPA ratio were significantly higher and the values of tPA were significantly lower in loculated than in nonloculated TBPE. These findings are in keeping with the results of the previous* in vitro* study [10] and raise the possibility that angiogenic cytokines, particularly VEGF, may attenuate pleural fibrinolytic activity by disrupting the balance of PAI-1 and tPA elaborated by endothelial and/or mesothelial cells and that the increase in VEGF is associated with the decrease in fibrinolytic activity and subsequent fibrin deposition and fluid loculation in TBPE.

The sequel of RPT of >10 mm may cause significant functional disturbance [25]. However, the predictors affecting the development of RPT in patients with TBPE remain elusive. Previous studies reported that the concentrations of C-reactive protein, IL-1, IL-8, tumor necrosis factor-*α*, transforming growth factor-*β*1, interferon-*γ*, and PAI-1 were significantly higher and the values of pH, glucose, and tPA were significantly lower in TBPE complicated with RPT than those without [6, 26–30]. Moreover, pleural fluid loculation or fibrin septation detected by chest US as an initial presentation may be of value in predicting the development or occurrence of RPT in TBPE following completion of anti-TB medication [6, 31]. A previous* in vivo* study also demonstrated that angiogenesis was required in the development of pleural fibrosis [11, 12].

In this study, fourteen patients who developed RPT at the end of follow-up presented initially with fluid loculation and had greater effusion size, higher effusion levels of VEGF, IL-8, PAI-1, and PAI-1/tPA ratio, and lower effusion levels of pH and tPA. Furthermore, the multivariate analysis demonstrated that larger effusion size or higher effusion level of VEGF was the independent risk factor for development of RPT. Our results also revealed that effusion VEGF >842 pg/mL, followed by effusion CXR score >62%, had optimal sensitivity and specificity to predict RPT in TBPE. At variance with the previous reports [6, 30], our study indicated that the presence of loculation did not increase the risk of pleural fibrosis. The discrepancy may be explained in part by the fact that all loculated TBPE patients in the present study received chest US-guided drainage and streptokinase irrigation, which may minimize the effect of effusion loculation on the occurrence of RPT [15]. Collectively, in agreement with the previous* in vivo* reports [11–13], our study indicated that the increased angiogenic activity in the pleural fluid might contribute to subsequent development of pleural fibrosis and signified the role of VEGF-related impaired fibrinolytic activity in the formation of RPT in TBPE.

A previous study demonstrated that administration of corticosteroids, in conjunction with anti-TB therapy, resulted in more rapid improvement in symptoms of fever and chest pain and in resolution of effusions in patients with TBPE, suggesting the beneficial effect of anti-inflammatory agents on clinical outcome [32]. However, to date, no effective medical treatment is available for preventing pleural fibrosis in TBPE [33]. The reason may be that another critical factor in pleural fibrosis is the formation of fibrinous neomatrix which results from disorder in fibrin turnover [8]. Transforming growth factor (TGF)-*β*, like VEGF [11, 12], could cause PAI-1/tPA imbalance and disordered fibrin turnover, and intrapleural injection with anti-TGF-*β* antibody has been shown to decrease pleural fibrosis in experimental empyema in rabbits [34]. Accordingly, the previous studies [32–34] and our results may justify further researches on the usefulness of anti-VEGF therapy for TBPE.

Taken together, the present study highlighted the pivotal role of VEGF in orchestration of inflammation, formation of pleural fluid, impairment of fibrinolysis, and development of residual fibrosis in TBPE. As prompted by the promising effect of targeted therapy with antiangiogenic agents on outcome of patients with metastatic colorectal cancer [35] and neovascular age-related macular degeneration [36], further preclinical and clinical trials are required to investigate the potential use of targeting VEGF as a therapeutic strategy adjunct to standard anti-TB treatment for TBPE.

## Figures and Tables

**Table 1:** Demographic and clinical data of the patients studied.

	All patients	Loculated TBPE	Nonloculated TBPE	*P* value^†^
	(*n* = 42)	(*n* = 26)	(*n* = 16)	
Male, *n* (%)	27 (64)	18 (69)	9 (56)	0.511
Age, yrs, mean ± SD	62 ± 21	61 ± 22	63 ± 21	0.824
Patients with comorbidities, *n* (%)^*^	28 (67)	18 (70)	10 (63)	0.742
Symptom onset to treatment, days, median (range)	10 (7–19)	10 (8–20)	10 (6–16)	0.547
Side of pleural effusion				
Right/left, *n* (%)	27/15 (64/36)	17/9 (65/35)	10/6 (63/37)	0.733

TBPE: uncomplicated parapneumonic effusion.

^*^Comorbidities including alcoholism, diabetes mellitus, neurologic, cardiac, respiratory, liver, and kidney diseases.

^†^For comparisons between loculated and nonloculated TBPE groups.

**Table 2:** Pleural effusion variables between loculated and nonloculated tuberculous pleural effusion.

	All patients	Loculated TBPE	Nonloculated TBPE	*P* value^†^
	(*n* = 42)	(*n* = 26)	(*n* = 16)	
Effusion CXR score, %, mean ± SD	53 ± 20	56 ± 21	43 ± 12	0.025

ADA, IU/L	99	108	86	0.100
	(66–185)	(82–203)	(59–149)	

pH value	7.30	7.27	7.36	<0.001
	(7.22–7.35)	(7.22–7.30)	(7.33–7.41)	

Glucose, mg/dL	116	98	120	0.038
	(75–138)	(75–118)	(99–142)	

Protein, g/L	5.0	4.8	5.2	0.089
	(4.1–5.3)	(4.1–5.2)	(4.2–5.6)	

LDH, IU/dL	307	387	210	0.002
	(229–533)	(287–723)	(154–388)	

Leukocyte count, cells/*μ*L	1598	1665	1437	0.449
	(963–3880)	(1330–3880)	(720–2000)	

PAI-1, ng/mL	114.6	138.5	105.5	<0.001
	(105.5–199.0)	(114.8–213.0)	(96.0–113.0)	

tPA, ng/mL	17.0	15.9	24.9	<0.01
	(10.0–23.0)	(8.4–20.5)	(14.4–28.7)	

PAI-1/tPA ratio	7.6	13.4	4.6	0.036
	(5.0–14.0)	(7.6–18.4)	(1.8–6.8)	

IL-8, pg/mL	365	419	167	<0.001
	(220–637)	(312–1442)	(79–395)	

VEGF, pg/mL	693	969	510	<0.001
	(499–1909)	(571–2054)	(161–713)	

TBPE: tuberculous pleural effusion; effusion CXR score: portion of hemithorax opacified by pleural effusion on posteroanterior chest radiograph; ADA: adenosine deaminase; LDH: lactate dehydrogenase; PAI-1: plasminogen activator inhibitor-1; tPA: tissue type plasminogen activator; IL-8: interleukin-8; VEGF: vascular endothelial growth factor.

Data are presented as median (IQR) unless specified.

^†^For comparisons between loculated and nonloculated TBP groups.

**Table 3:** Correlation among angiogenic cytokines, fibrinolytic parameters, pleural fluid characteristics, and effusion CXR scores.

	pH	Glucose	LDH	Leukocyte count	PAI-1	tPA	PAI-1/tPA ratio	Effusion CXR score
Loculated TBPE (*n* = 26)								
IL-8	−0.42^*^	−0.40^*^	0.39^*^	0.36	0.28	−0.40^*^	0.42^*^	0.21
VEGF	−0.57^†^	−0.58^†^	0.49^*^	0.25	0.77^‡^	−0.53^†^	0.76^‡^	0.63^‡^
Effusion CXR score	−0.14	−0.12	−0.18	0.13	0.39^*^	−0.22	0.12	—
Nonloculated TBPE (*n* = 16)								
IL-8	−0.46	−0.48	0.55^*^	0.28	0.24	−0.62^†^	0.59^*^	0.32
VEGF	−0.52^*^	−0.56^*^	0.67^†^	0.17	0.59^*^	−0.44^*^	0.43^*^	0.47^*^
Effusion CXR score	−0.22	−0.29	−0.11	0.29	0.21	−0.11	0.10	—

See Table 2 for definition of the abbreviations.

^*^Correlation is statistically significant at the level of 0.05.

^†^Correlation is statistically significant at the level of 0.01.

^‡^Correlation is statistically significant at the level of 0.001.

**Table 4:** Pleural fluid variables and pulmonary function in patients with or without development of residual pleural thickening (RPT).

	RPT (+)	RPT (−)	P value
	(n = 14)	(n = 28)	
Effusion status			
Effusion CXR score, %, mean ± SD	71 ± 20	44 ± 12	<0.001
Loculation, n (%)	14 (100)	0 (0)	0.002
Pleural fluid			
pH value	7.27 (7.22–7.30)	7.35 (7.25–7.39)	0.013
Glucose, mg/dL	100 (73–140)	106 (76–129)	0.947
LDH, IU/dL	328 (229–666)	289 (154–532)	0.126
Leukocyte count, cells/μL	2840 (1521–4410)	1437 (720–2000)	0.028
PAI-1, ng/mL	143.1 (111.1–208.0)	113.7 (78.0–122.5)	0.043
tPA, ng/mL	10.4 (8.2–12.7)	15.8 (4.8–21.0)	0.028
PAI-1/tPA ratio	11.7 (5.0–16.7)	5.2 (2.9–8.4)	<0.001
IL-8, pg/mL	419 (312–985)	248 (96–502)	0.025
VEGF, pg/mL	2054 (1909–3387)	516 (274–693)	<0.001
FVC, % predicted			
At 6 months	74 (73–75)	80 (79–81)	<0.001

See Table 2 for definition of the abbreviations. RPT: residual pleural thickening ≥10** **mm shown on CXR at the end of 6-month follow-up; FVC: forced vital capacity.

Data are presented as median (IQR) unless specified.

**Table 5:** Multivariate logistic regression analyses of factors associated with residual pleural thickening (RPT).

	Odds ratio	95% CI	*P* value
Effusion status			
Effusion CXR score, %	1.01	1.00–1.02	0.02
Loculation	1.00	0.99-1.00	0.99
Pleural fluid			
pH value	1.01	0.98–1.04	0.52
Leukocyte count, cells/*μ*L	1.00	0.99-1.00	0.87
PAI-1, ng/mL	0.99	0.97–1.03	0.76
tPA, ng/mL	1.00	0.99-1.00	0.99
PAI-1/tPA ratio	1.01	0.98–1.04	0.52
IL-8, pg/mL	1.00	0.99-1.00	0.93
VEGF, pg/mL	1.28	1.06–1.51	0.01

See Table 2 for definition of the abbreviations. CI: confidence interval.

## Abbreviations

CT: Computed tomography
CXR: Chest radiography
IL-8: Interleukin-8
PAI-1: Plasminogen activator inhibitor-1
PE: Pleural effusion
RPT: Residual pleural thickening
tPA: Tissue type plasminogen activator
TB: Tuberculosis
TBPE: Tuberculous pleural effusion
US: Ultrasonography
VEGF: Vascular endothelial growth factor.

## References

[1] Dye C., Williams B. G. (2010). The population dynamics and control of tuberculosis. *Science*.

[2] Porcel J. M. (2009). Tuberculous pleural effusion. *Lung*.

[3] Antony V. B. (2003). Immunological mechanisms in pleural disease. *European Respiratory Journal*.

[4] Mohammed K. A., Nasreen N., Hardwick J., Logie C. S., Patterson C. E., Antony V. B. (2001). Bacterial induction of pleural mesothelial monolayer barrier dysfunction. *The American Journal of Physiology—Lung Cellular and Molecular Physiology*.

[5] Broaddus V. C., Boylan A. M., Hoeffel J. M. (1994). Neutralization of IL-8 inhibits neutrophil influx in a rabbit model of endotoxin-induced pleurisy. *Journal of Immunology*.

[6] Chung C. L., Chen C. H., Sheu J. R., Chen Y. C., Chang S. C. (2005). Proinflammatory cytokines, transforming growth factor-*β*1, and fibrinolytic enzymes in loculated and free-flowing pleural exudates. *Chest*.

[7] Bithell T. C. (1993). *Wintrobe’s Clinical Hematology*.

[8] Idell S., Girard W., Koenig K. B., McLarty J., Fair D. S. (1991). Abnormalities of pathways of fibrin turnover in the human pleural space. *The American Review of Respiratory Disease*.

[9] Idell S., Mazar A. P., Bitterman P., Mohla S., Harabin A. L. (2001). Fibrin turnover in lung inflammation and neoplasia. *The American Journal of Respiratory and Critical Care Medicine*.

[10] Pepper M. S., Ferrara N., Orci L., Montesano R. (1991). Vascular endothelial growth factor (VEGF) induces plasminogen activators and plasminogen activator inhibitor-1 in microvascular endothelial cells. *Biochemical and Biophysical Research Communications*.

[11] Guo Y. B., Kalomenidis I., Hawthorne M., Parman K. S., Lane K. B., Light R. W. (2005). Pleurodesis is inhibited by anti-vascular endothelial growth factor antibody. *Chest*.

[12] Teixeira L. R., Vargas F. S., Acencio M. M. P. (2011). Blockage of vascular endothelial growth factor (VEGF) reduces experimental pleurodesis. *Lung Cancer*.

[13] Ribeiro S. C. C., Vargas F. S., Antonangelo L. (2009). Monoclonal anti-vascular endothelial growth factor antibody reduces fluid volume in an experimental model of inflammatory pleural effusion. *Respirology*.

[14] Chung C. L., Hsiao S. H., Hsiao G., Sheu J. R., Chen W. L., Chang S. C. (2013). Clinical Importance of Angiogenic Cytokines, Fibrinolytic Activity and Effusion Size in Parapneumonic Effusions. *PLoS ONE*.

[15] Chung C. L., Chen C. H., Yeh C. Y., Sheu J. R., Chang S. C. (2008). Early effective drainage in the treatment of loculated tuberculous pleurisy. *European Respiratory Journal*.

[16] Jiménez Castro D., Díaz G., Pérez-Rodríguez E., Light R. W. (2003). Prognostic features of residual pleural thickening in parapneumonic pleural effusions. *European Respiratory Journal*.

[17] Cheng D. S., Rodriguez R. M., Perkett E. A. (1999). Vascular endothelial growth factor in pleural fluid. *Chest*.

[18] Economidou F., Antoniou K. M., Tzanakis N., Sfiridaki K., Siafakas N. M., Schiza S. E. (2008). Angiogenic molecule Tie-2 and VEGF in the pathogenesis of pleural effusions. *Respiratory Medicine*.

[19] Kiropoulos T. S., Kostikas K., Gourgoulianis K. I. (2005). Vascular endothelial growth factor levels in pleural fluid and serum of patients with tuberculous pleural effusions. *Chest*.

[20] Alatas F., Alatas Ö., Metintas M., Özarslan A., Erginel S., Yildirim H. (2004). Vascular endothelial growth factor levels in active pulmonary tuberculosis. *Chest*.

[21] Grove C. S., Lee Y. C. G. (2002). Vascular endothelial growth factor: the key mediator in pleural effusion formation. *Current Opinion in Pulmonary Medicine*.

[22] Wu Y., Zhang Q., Ann D. K. (2004). Increased vascular endothelial growth factor may account for elevated level of plasminogen activator inhibitor-1 via activating ERK1/2 in keloid fibroblasts. *The American Journal of Physiology—Cell Physiology*.

[23] Fukumoto T., Matsukawa A., Yoshimura T. (1998). IL-8 is an essential mediator of the increased delayed-phase vascular permeability in LPS-induced rabbit pleurisy. *Journal of Leukocyte Biology*.

[24] Alemán C., Alegre J., Monasterio J. (2003). Association between inflammatory mediators and the fibrinolysis system in infectious pleural effusions. *Clinical Science*.

[25] Candela A., Andujar J., Hernández L. (2003). Functional sequelae of tuberculous pleurisy in patients correctly treated. *Chest*.

[26] de Pablo A., Villena V., Echave-Sustaeta J., Encuentra A. L. (1997). Are pleural fluid parameters related to the development of residual pleural thickening in tuberculosis?. *Chest*.

[27] Hua C. C., Chang L. C., Chen Y. C., Chang S. C. (1999). Proinflammatory cytokines and fibrinolytic enzymes in tuberculous and malignant pleural effusions. *Chest*.

[28] Kunter E., Ilvan A., Kilic E. (2002). The effect of pleural fluid content on the development of pleural thickness. *International Journal of Tuberculosis and Lung Disease*.

[29] Lin F. C., Chen Y. C., Chen F. J., Chang S. C. (2005). Cytokines and fibrinolytic enzymes in tuberculous and parapneumonic effusions. *Clinical Immunology*.

[30] Gerogianni I., Papala M., Tsopa P. (2008). Could IFN-*γ* predict the development of residual pleural thickening in tuberculous pleurisy?. *Monaldi Archives for Chest Disease—Pulmonary Series*.

[31] Lai Y. F., Su M. C., Weng H. H., Wu J. T., Chiu C. T. (2009). Sonographic septation: a predictor of sequelae of tuberculous pleurisy after treatment. *Thorax*.

[32] Lee C. H., Wang W. J., Lan R. S., Tsai Y. H., Chiang Y. C. (1988). Corticosteroids in the treatment of tuberculous pleurisy. A double-blind, placebo-controlled, randomized study. *Chest*.

[33] Huggins J. T., Sahn S. A. (2004). Causes and management of pleural fibrosis. *Respirology*.

[34] Kunz C. R., Jadus M. R., Kukes G. D., Kramer F., Nguyen V. N., Sasse S. A. (2004). Intrapleural injection of transforming growth factor-*β* antibody inhibits pleural fibrosis in empyema. *Chest*.

[35] Pavlidis E. T., Pavlidis T. E. (2013). Role of bevacizumab in colorectal cancer growth and its adverse effects: a review. *World Journal of Gastroenterology*.

[36] Chiang A., Regillo C. D. (2011). Preferred therapies for neovascular age-related macular degeneration. *Current Opinion in Ophthalmology*.

